# Anaerobes and Toxins, a Tradition of the Institut Pasteur

**DOI:** 10.3390/toxins15010043

**Published:** 2023-01-05

**Authors:** Michel R. Popoff, Sandra Legout

**Affiliations:** 1 Bacterial Toxins, Institut Pasteur, 75724 Paris, France; 2 Centre de Ressources en Information Scientifique, Institut Pasteur, 75015 Paris, France

**Keywords:** Pasteur, anaerobe, toxin, anatoxin, vaccine

## Abstract

Louis Pasteur, one of the eminent pioneers of microbiology, discovered life without oxygen and identified the first anaerobic pathogenic bacterium. Certain bacteria were found to be responsible for specific diseases. Pasteur was mainly interested in the prevention and treatment of infectious diseases with attenuated pathogens. The collaborators of Pasteur investigated the mechanisms of pathogenicity and showed that some bacterial soluble substances, called toxins, induce symptoms and lesions in experimental animals. Anaerobic bacteriology, which requires specific equipment, has emerged as a distinct part of microbiology. The first objectives were the identification and taxonomy of anaerobes. Several anaerobes producing potent toxins were associated with severe diseases. The investigation of toxins including sequencing, mode of action, and enzymatic activity led to a better understanding of toxin-mediated pathogenicity and allowed the development of safe and efficient prevention and treatment (vaccination with anatoxins, specific neutralizing antisera). Moreover, toxins turned out to be powerful tools in exploring cellular mechanisms supporting the concept of cellular microbiology. Pasteurians have made a wide contribution to anaerobic bacteriology and toxinology. The historical steps are summarized in this review.

## 1. Introduction

Louis Pasteur was one of the pioneers of microbiology. He showed the role of bacteria in the fermentation processes and discovered that some bacteria can live without oxygen. Bacteria were found also to be responsible for specific diseases. L. Pasteur described the first anaerobic pathogenic bacterium. His main interest was the prevention and treatment of infectious diseases based on attenuated pathogens. Then, the collaborators of L. Pasteur, and the community of scientists at Institut Pasteur and outside developed anaerobic bacteriology and investigated the mechanisms of bacterial pathogenicity. Soluble factors secreted by certain bacteria were found to be involved in the onset of symptoms and lesions and were termed toxins. This short review summarizes the historical aspects of anaerobic bacteriology and toxinology developed by Pasteurians and collaborators. The data are presented in chronological order.

## 2. “Poisons” before the Pasteur’s Era

Poisons have been known from the most ancient times. Very early on, humans had to deal with dangerous substances produced by certain plants or secreted by some animals. The knowledge of these dangers based on cumulative observations of the fortuitous ingestion of certain plants/mineral compounds and accidental encounters with venomous animals was likely transmitted to individuals of the clan/tribe and then from generation to generation. An initial use of poisons was probably for hunting and fishing, and poisons have also been widely employed for war and criminal purposes. It was speculated that hunters of the Mesolithic era (about 9000–1000 BC) used poisonous weapons. Early archeological evidence is from Ancient Egypt (about 2000 BC). Arrows were found to contain poisonous substances inducing strophanthin- or curare-like symptoms in mice, probably from plant origin [1]. In the Antiquity period, poisons are mentioned in several texts such as those from Homer (The Odyssey and The Iliad, about 800 BC), Hippocrates, Democritus, who investigated the effects of several poisons, and Aristotle, who described arrow poisons [1,2]. The term “toxicon” derives from the Greek word τοξικον formed from the word τοξον meaning bow. Thus, toxicon designated arrow poisons. All drugs including harmful substances or substances used for treating diseases were designated as “pharmakon” or “pharmaka”. The Romans used the word “toxicum” for any poison [1,2]. The terms poison and potion derive from the Latin word “potionem” meaning drink, as they designate substances generally absorbed by the oral route. Then, poison was used for the designation of harmful substances and potions for medicines.

The most ancient documents related to poisons and medicines are from the Sumerian (as early as 4000 B.C.E.) and Egyptian (1500 B.C.E.) civilizations [2]. The distinction between poison and medicine is sometimes subtle. Paracelsus, in the early Renaissance period (1493–1541), argued that “the dose makes the poison” [2].

In the 19th century, putrefaction products (pus material, putrid meat, putrid vegetables) were found to be lethal when injected into animals (reviewed in [3]). The origin and nature of the “putrid poisons” (Peter Panum 1856) were a matter of debate. They were supposed to derive from the decomposition of organic material or from a bacterial process. The term “ptomaïnes” (Francesco Selmi 1878) was used to designate cadaveric alkaloids. Ludwig Brieger (1885–1886) further characterized putrescine and cadaverine and showed that bacteria are able to produce specific toxic compounds. He introduced the specific name “toxins” for bacterial toxic substances [4].

## 3. Anaerobes and Toxins in Pasteur’s Era

The major contribution of Louis Pasteur and the German physician Robert Koch is the demonstration that specific bacteria are responsible for specific diseases. First, Casimir J. Davaine isolated and R. Koch identified the “bactéridie charbonneuse”, then called *Bacillus anthracis*, as the causative agent of anthrax [5]. At this period, the mechanism of bacterial pathogenicity was obscure. It was questionable whether the whole live bacteria or only a fraction of the bacteria were required to promote the symptoms and lesions of the disease. R. Koch (1883) who isolated the agent of cholera (*Vibrio cholerae*) suggested that the disease was due to a “poison” produced by the bacteria. The preliminary observations of the Italian physician Arnaldo Cantani (1886) showed that the cultures of *V. cholera* injected in dogs were toxic. Independently, Richard Pfeiffer (1892–1894) and Nicolas Gamaleïa (1892) confirmed the production of the cholera toxin by the agent of cholera [3,6]. N. Gamaleïa was a Russian microbiologist who worked in the laboratory of L. Pasteur (1886) and with Elie Metchnikoff. Friedrich Löffler (1884), a collaborator of R. Koch, isolated the causative agent of diphtheria that was identified one year before by the German medical doctor Edwin Klebs. Löffler argued that the tissue damages result from a toxic compound synthesized by the diphtheria bacillus that diffuses from the localized infection site throughout the host [3,7].

In 1888, Emile Roux (collaborator of L. Pasteur from 1878 to 1888 and then-Director of the Institut Pasteur (1904–1933)) and Alexandre Yersin (collaborator of L. Pasteur and E. Roux from 1885–1890) at the Institut Pasteur, Paris, demonstrated the presence of the diphtheria toxin in culture filtrates of the diphtheria bacillus that was able to induce similar lesions to those observed in natural disease and death in experimental animals [8]. The cholera toxin and diphtheria toxin were the two first bacterial toxins that were identified. A. Cantini and R. Pfeiffer distinguished between endotoxins that are linked to the bacterial cells and exotoxins that are released in the culture medium [6].

Although L. Pasteur recognized the existence of bacterial poisons, he was doubtful about the role of toxins in diseases [9]. He was focused on the prevention of infectious diseases by attenuated bacteria that he called vaccination in honor of Edward Jenner who prevented smallpox through the inoculation of cowpox. L. Pasteur discovered that an old culture of the causative agent of fowl cholera (*Pasteurella multocida*) was avirulent and can protect against the inoculation of virulent challenges. This was the first bacterial vaccine. Then, he was interested in the prevention of anthrax which was a severe disease widely spread in cattle and sheep. He tried several protocols to inactivate *B. anthracis*. In 1881, in the famous experiment at Pouilly Le-Fort, L. Pasteur succeeded in demonstrating the efficacy of vaccination against anthrax with an attenuated *B. anthracis* strain and became a great celebrity. However, L. Pasteur was not the first discoverer of the anthrax vaccine. A few months earlier, Henry Toussaint, a French scientist, and William Smith Greenfield, a British veterinarian, showed that attenuated *B. anthracis* can protect against anthrax [5,9,10]. It is noteworthy that the two first vaccines against bacterial infectious diseases were performed with toxigenic bacteria that were attenuated in the production of toxins. However, the mechanism of vaccination remained mysterious. L Pasteur admitted that a bacterial soluble substance might induce the protection, but he was more convinced that attenuated bacteria act by competition with pathogens by depleting the host of essential nutrients required for bacterial viability and growth (discussed in this issue [9]).

During his studies on fermentation (1857–1877), L. Pasteur discovered life without oxygen. First, he identified that sugar fermentation results from an aerobic yeast alcohol fermentation followed by an anaerobic yeast lactic fermentation. Then, in 1861, he found that butyric fermentation was associated with a motile, spore-forming microorganism able to grow in the absence of free oxygen. He called this “infusoire”, “butyric vibrio”, probably from the *Clostridium butyricum/Clostridium beijerinckii/Clostridium acetobutylicum* group [9,11]. In 1863, L. Pasteur distinguished aerobic and anaerobic microorganisms, anaerobes being microorganisms growing in the absence of air and for which air is lethal [11]. In 1865, L. Pasteur and his collaborator Jules Joubert identified the first pathogenic anaerobe from a sheep that died of septicemia supposed to be anthrax. In 1877, they succeeded in isolating and cultivating the microorganism in anaerobic conditions and termed it “septic vibrio” [12].

**Figure 1:**
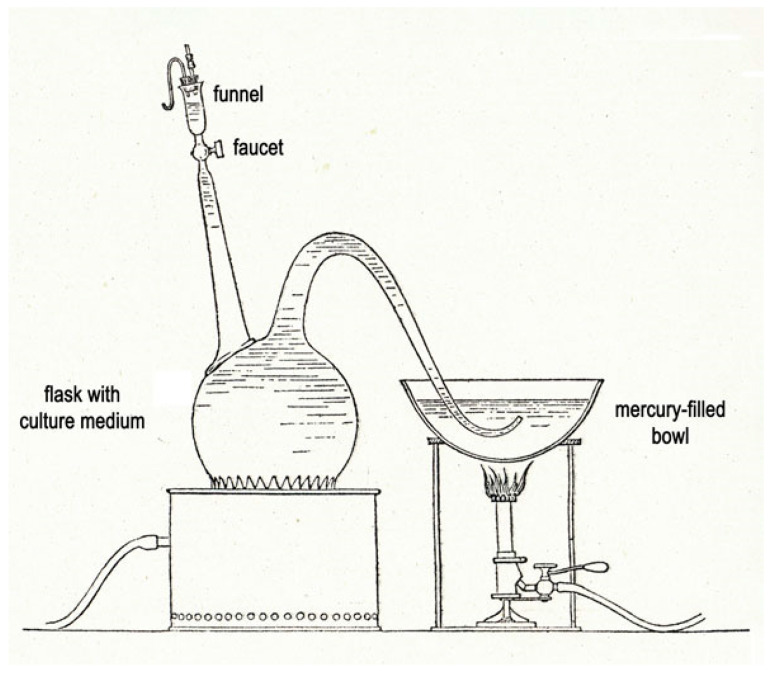
Device developed by L. Pasteur for anaerobic culture [14] (Institut Pasteur/Musée Pasteur). A 6 L flask containing the culture medium is boiled for at least 30 min to eliminate the dissolved oxygen. After cooling at 25–30 °C the curved tube is connected to a mercury-filled bowl. The funnel is flushed with carbon dioxide and simultaneously filled with 10 mL culture inoculum. The flask is inoculated by opening the faucet with precaution to keep a small volume of inoculum in the funnel, avoiding the introduction of air.

L. Pasteur developed a basic method for culture in anaerobic conditions. A glass balloon containing liquid culture medium was boiled to release all the air, and the glass tapered end was closed with a flame [13,14] (Figure 1). In 1887, E. Roux described several techniques for culturing microorganisms in anaerobiosis [15]. From around 1895, a novel technique without vacuum or air replacement was introduced by Adrien Veillon (1864–1931) when he was a young scientist in the laboratory of Jacques-Joseph Grancher, Director of the “Hôpital des Enfants Malades” (Paris) and collaborator of L. Pasteur. This method consisted of deep agar in long glass tubes (Veillon’s tubes). A. Veillon investigated the microorganisms responsible for suppuration. He showed that many anaerobic bacteria are saprophytes in the oral cavity of humans and distinguished between the endogenous and exogenous microflora [16]. In 1900, A. Veillon joined the laboratory of the “Microbie technique” of E. Roux, and one year later, he was a physician at the hospital of Institut Pasteur. During the First World War, he was involved in the treatment and prevention of war wounds, notably gas gangrene due to *C. perfringens.* He amended the fight against gangrene by creating laboratories of bacteriology in military hospitals of surgery [17].

## 4. Period 1900–1940: The “Microbie Technique”

During the period of about 1904–1914, several scientists worked on anaerobes and bacterial toxins in the service of the “Microbie technique” of E. Roux: Jean Binot, Edouard Dujardin-Baumetz, Constatin Levaditi, René Legroux, Auguste Charles Marie, Victor Morax, Maurice Nicolle, Aleaxandre Salimbeni, Adrien Veillon, and Michel Weinberg.

**Figure 2:**
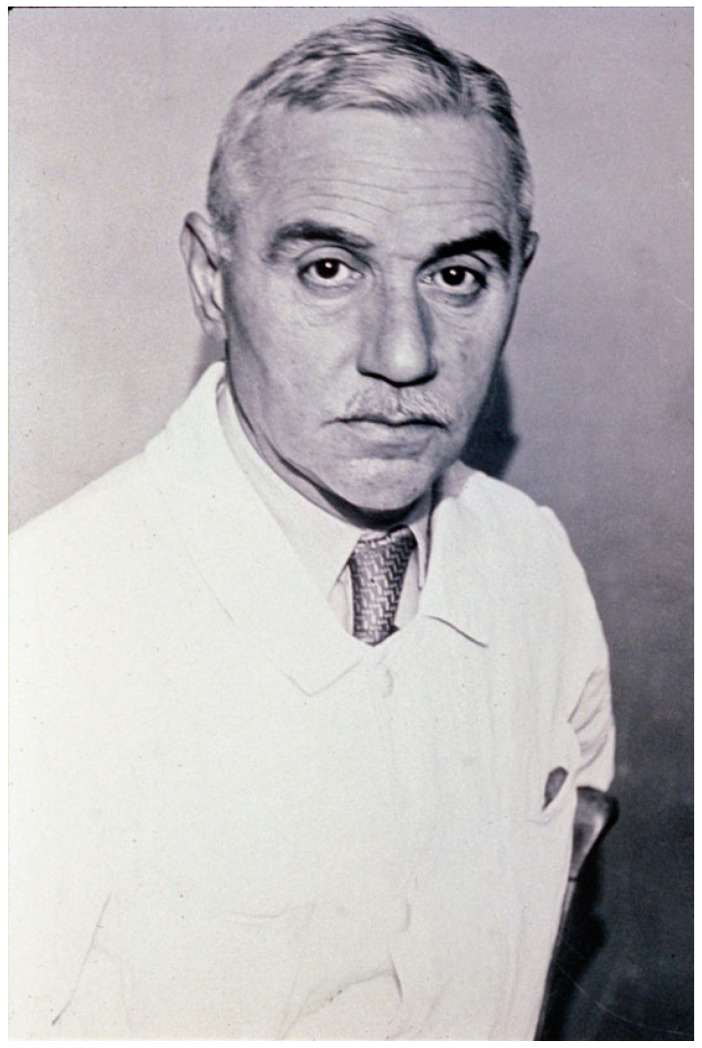
René Legroux (1877–1951) portrait of about 1930 (Institut Pasteur/Musée Pasteur). R. Legroux investigated human botulism during the Second World War and prepared botulinum anatoxins and anti-sera.

The agent of botulism was identified by Emile van Ermengem in Belgium (*Bacillus botulinus*, then *Clostridium botulinum*) [18]. In France, the first reported botulism outbreak in humans was provided by the medical doctor Octave Du Mesnil in 1875 [19]. Human botulism was rare in France until the Second World War [20]. During the period of 1940–1944, the incidence of botulism was high as reported by R. Legroux and collaborators at the Institut Pasteur, mainly due to poor hygiene in the preparation of homemade preserved food [21,22,23] (Figure 2). In 1926, René Legroux and André-Pierre Marie prepared concentrated botulinum toxin. Unfortunately, A.-P. Marie died from botulism, probably by eye contamination with a tiny particle of concentrated and dried toxin [24,25]. Another collaborator, Colette Jeramec, was also contaminated, but less severely. In 1934, R. Legroux, with Lucien Second, succeeded in producing liquid botulinum anatoxin and anti-botulinum serum in horses, which was used for the treatment of patients with botulism during the Second World War [24]. Serotherapy against botulism was further analyzed in guinea pigs [26]. Legroux and collaborators investigated experimental botulism in rabbits and horses. Notably, they explored the influence of the route of toxin inoculation on the induction of the disease [27,28].

**Figure 3:**
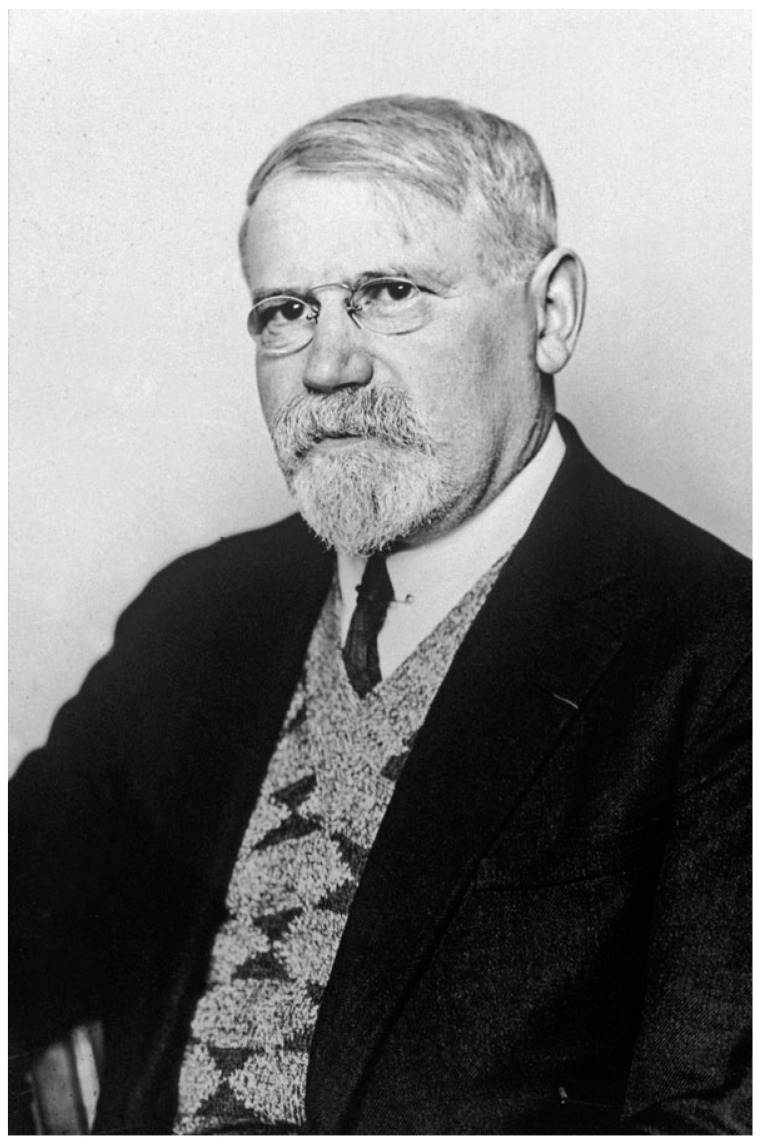
Michel Weinberg’s (1868–1940) portrait of about 1930. (Institut Pasteur/Musée Pasteur). M. Weinberg studied the agents of gangrene and wrote with B. Ginsbourg a book on the taxonomy of anaerobes.

Michel Weinberg (1866–1940), a Russian physician born in Odessa, performed his medical studies in Paris (1892–1898), and he joined the Metchnikoff’s service of “Microbie Morphologique” at the Institut Pasteur in 1900 (Figure 3). He studied the role of helminths in the translocation of bacterial pathogens through the intestinal mucosa, notably in the onset of appendicitis. During the First World War, he worked on the agents of gangrenes that affected numerous wounded soldiers. He found that the microbes responsible for gangrenes are diverse, and he identified novel species such as *Bacillus oedematiens* (*Clostridium novyi*), *B. fallax* (*C. fallax*), and *B. aerofetidus*. In 1918, he published a book with Pierre Seguin on gangrenes, in 1927 a book with B. Ginsbourg on the taxonomy of anaerobes and their role in pathology, and an updated one on anaerobes with R. Nativelle and A. R. Prévot in 1937 [29,30,31]. Then, he developed sera against the agents of gangrenes that were used by numerous surgeons. His work has been the subject of abundant publications in scientific journals [32].

The agent of tetanus was first identified by Arthur Nicolaier in 1884 and successfully isolated and cultivated by Shibasaburo Kitasato in the Emil von Behring laboratory (Berlin) in 1889 [9]. This agent was found to be an anaerobic bacterium with typical round terminal spores giving the appearance of drumsticks (*Bacillus tetani*, then *Clostridium tetani*), and it was demonstrated that the symptoms of tetanus are induced by a toxin (Tetanus toxin, TeNT) released in culture filtrates [9]. Von Behring and Kitasato showed that the sera from immunized animals against tetanus toxin were protective [33]. E. Roux and Louis Vaillard (1893), a French military physician, further investigated tetanus in experimental animals and developed iodinated TeNT as an immunogen. They confirmed that sera against TeNT can prevent the disease and are curative when administrated soon after the onset of symptoms [34]. Auguste-Charles Marie (1864–1935), a French physician and scientist, joined the Institut Pasteur in the service of E. Metchnikoff and then of E. Roux. He analyzed the trafficking of TeNT in rabbits, mice, and guinea pigs. He showed with Victor Morax, a Swiss physician and biologist who joined the laboratory of Microbie Technique, that the toxin, when administrated by the intravenous route, remains only a short time in the blood circulation and is trapped by the nervous system. In 1902–1903, A-C. Marie and V. Morax suggested that TeNT is transported by the peripheral neurons to the central nervous system [35,36]. The retrograde axonal transport of TeNT by motorneurons to the central nervous system was experimentally confirmed by several scientific teams in the period 1955–1979 (reviewed in [37,38]). More recently, Schiavo et al. further analyzed the movement of TeNT in cultured neurons [39].

A great step in the preparation of antigens was accomplished by the Pasteurian Gaston Ramon (1886–1963), a French veterinarian and biologist, who was in charge of the production of antisera in horses at the annex of Garches. In 1923, G. Ramon found that diphtheria toxin treated with low doses of formalin was a safe and potent immunogen and coined the term “anatoxin” [40]. The same procedure was successfully developed to prepare the tetanus anatoxin in 1925 [9,41]. Ramon observed a flocculation phenomenon when diphtheria toxin was mixed with a corresponding antiserum and developed an in vitro assay for the titration of antigens by flocculation [42,43]. The flocculation method for the titration of tetanus anatoxin is still a reference method in the Pharmacopeia.

The scientific activity at the Institut Pasteur was spread in several laboratories without specific denominations and not organized in scientific departments that were set up in 1967. In the scientific report of 1934 (ARCH. DUJ.C.1), E. Roux mentioned that the activity of M. Weinberg and his students on anaerobic microbes was of great importance and that they succeeded in obtaining anatoxins of botulism and gas gangrene agents based on the method developed by G. Ramon. It was recognized that the works of M. Weinberg were determinant for obtaining antigangrene sera that were used during the First World War. In the re-organization of the Institut Pasteur in 1934, a laboratory dedicated to anaerobes was created and called the “Service des microbes anaérobies”. It was directed by M. Weinberg until his death in 1940. In 1936, an extension of the laboratory spaces was attributed to M. Weinberg for his activities on anaerobes.

It was noted in the report on the Institut Pasteur reorganization of 1942 by René Dujarric de la Riviere, Secretary general of the Institut Pasteur (ARCH. DUJ.C.1), that “since the nice works of Pasteur, followed by those of Veillon and Weinberg, the study of anaerobes has taken rightly an important place at Institut Pasteur. The species identification by cultural aspects, lesions induced in animals, immunological properties are now completed with biochemical investigations”.

## 5. Period 1941–1968: The “Service des Anaérobies”

**Figure 4:**
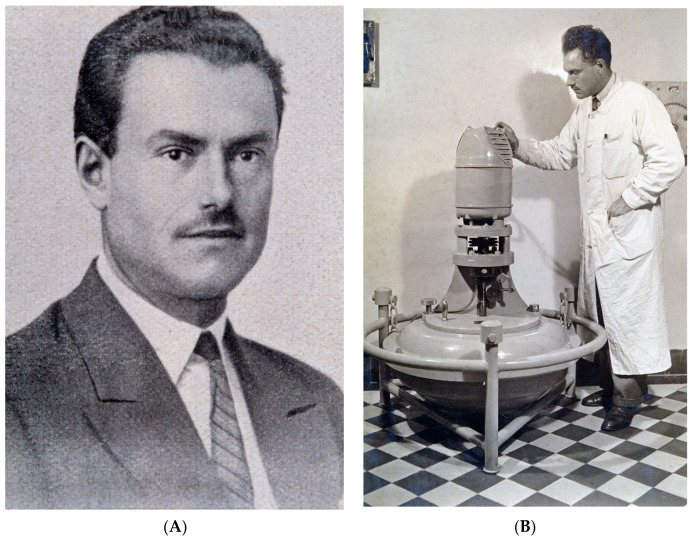
André-Romain Prévot (1894–1982) (**A**) portrait of about 1930–1935. A.-R. (**B**) Prévot in his laboratory monitoring a continuous feed centrifuge. A.-R. Prévot developed anaerobic bacteriology and taxonomy, and he investigated botulism in France (Institut Pasteur/Musée Pasteur).

André-Romain Prévot (1894–1982) obtained a certificate in mineralogy at the Faculty of Sciences of Lille and then studied medicine in Paris (Figure 4). During the First World War, as an auxiliary military officer, he was confronted with many cases of gangrene and tetanus. In 1924, he wrote his medical thesis on anaerobic streptococci and his PhD on anaerobic cocci in 1933. A.-R. Prévot joined the laboratory of M. Weinberg dedicated to anti-gangrene serotherapy at the Institut Pasteur in 1922. He was named the deputy head of the laboratory of tetanus in 1939 and the head of the Anaerobe Laboratory (Service des Anaérobies) in 1941. A.-R. Prévot developed a specific laboratory on anaerobes until his retirement in 1966 [44]. A.-R. Prévot brought a great contribution to the taxonomy and physiology of anaerobes. He analyzed the respiratory types of bacteria and distinguished seven groups from strict anaerobic to strict aerobic bacteria. The bacterial classification was based on cultural, morphological, and biochemical properties. He isolated and characterized numerous novel anaerobe species. In addition to his numerous scientific publications, he wrote two voluminous books on anaerobes, one already mentioned by M. Weinberg and R. Nativelle in 1937 and another one with André Turpin and Paul Kaiser in 1967 which were reference books in the classification of these bacteria, as well as 13 other books on the biology of anaerobes, their metabolism, and their role in pathology and in the environment [31,45]. A.-R. Prévot was a member of the international committee of bacterial nomenclature, and he contributed actively to the definition of bacterial species and their classification. In the 1940s–1960s, the Institut Pasteur was recognized as an international reference center for the identification of anaerobes. Walter Edward Cladek Moore (1927–1996), a US microbiologist, was interested in anaerobic bacteria. W. E. Moore visited twice the service of A.-R. Prévot at the Institut Pasteur in the 1960s, and A.-R. Prévot gave him anaerobe samples from his entire collection. W. E. Moore took away about 2000 strains and created the anaerobe laboratory at the Virginia Polytechnic Institute, Blacksburg, which was the reference center for anaerobes in the US until his death in 1996 [46]. W. E. Moore with Lillian V. Holdeman and Elizabeth P. Cato edited the Anaerobe Laboratory Manual which was a recognized reference in the field of anaerobe identification [47].

A.-R. Prévot and collaborators identified the first cases of botulism in cattle and horses due to *C. botulinum* C and D in France [48,49]. They investigated experimental botulism in horses and developed an anatoxin against botulinum toxins C and D for animal vaccination [50]. Moreover, they described the first identification of human botulism type E in France [51] and an exceptional outbreak of human botulism type D in Chad which is the unique human botulism of this type reported in the literature [52,53]. Indeed, *C. botulinum* D is essentially involved in animal botulism. It is noteworthy that the strain isolated from this atypical outbreak and characterized by A.-R. Prévot is recognized as the reference strain of *C. botulinum* D [52,54].

In 1960, the Service des Anaérobies managed by A.-R. Prévot and M. Raynaud encompassed 20 laboratories with animal facilities, industrial production, and research laboratories in the Institut Pasteur, Paris, and the annexes of Garches.

Among the Pasteurian scientists working on anaerobes and toxins during this period, we have to mention Marcel Raynaud, André Turpin, Edgar-Hans Relyveld, and Marcel Rouyer.

Marcel Raynaud (1911–1974), a medical doctor (1940) and PhD (1946) on “*Clostridium sporogenes* soluble toxic substances”, joined the Institut Pasteur in 1942 in the biochemical laboratory of M. Macheboeuf and then the “Service des anaérobies” of A.-R. Prévot. In 1947, he became the head of the bacteriology and immunology laboratory. M. Raynaud developed multiple activities in bacteriology and immunology including protein toxins, endotoxins, toxin–antitoxin reactions, and horse immunoglobulins. Notably, he contributed to the production/purification of TeNT and the characterization of the TeNT anatoxin [55,56,57].

André Turpin (1920–1977), a French Doctor of Pharmacy, joined the laboratory of tetanus in Garches in 1947. He was the head of the laboratory of tetanus in 1964, which was renamed the laboratory of anaerobic toxins in 1970. A. Turpin performed the production of TeNT as well as of other toxins from anaerobes and collaborated with B. Bizzini and M. Raynaud on the deciphering of the TeNT structure [55,58,59,60] and on the study of anaerobes with A.-R. Prévot [45].

E.-H. Relyveld, a French scientist, spent his entire career (1952–1988) at the Institut Pasteur Garches where he was involved in the preparation of toxins and anatoxins and the production of vaccines. In addition to his activity on diphtheria toxin and staphylococcal toxins, he participated with M. Raynaud and A. Turpin in the preparation of TeNT and anti-tetanus serum [56,61].

Marcel Rouyer (1898–1981), a French medical doctor, entered the Institut Pasteur in 1940 and was successively the head of laboratory (1946) and the head of service (1956). M. Rouyer managed the laboratory of tetanus, then the laboratory of diphtheria, and finally the “Service des Anaérobies” from 1967 to 1968 following the retirement of A.-R. Prévot.

## 6. Period 1969–2000s: Towards the Genetics of Anaerobes and Molecular Mode of Action of Toxins

Madeleine Sebald (1930–) supported her medical thesis in 1957 and PhD in 1962 on the taxonomy of Gram-negative anaerobes. She joined the Service des Anaérobies of A.-R. Prévot in 1957 as a trainee and then a research assistant. From 1969 to 1996, M. Sebald was the Director of the Laboratory of Anaerobes, which was renamed the Unit of Anaerobes in 1977. M. Sebald markedly contributed to the taxonomy of anaerobes, more especially of Gram-negative anaerobes by introducing DNA-based methods such as GC% content that were novel approaches at this period. Then, M. Sebald was interested in the physiology and genetics of anaerobes including sporulation and spore germination in some *Clostridium* species. Thereby, she investigated the solventogenesis in *Clostridium acetobutylicum*, which regained industrial interest for the production of solvents from biomasses in 1981 after the first oil shock.

The genetic resistance to antibiotics has been analyzed in clostridia and in *Bacteroides fragilis*. Resistance to tetracycline, chloramphenicol, and macrolides has been determined to be supported by plasmids or transposons in *C. perfringens* and in *B. fragilis*. For the first time, the transferability of plasmids carrying antibiotic resistance genes between anaerobes such as between *C. perfringens* strains, *C. perfringens*/*Clostrioides difficile* (formerly *Clostridium difficile*), and *B. fragilis* strains has been demonstrated [62,63,64]. This opened the way to build shuttle vectors as tools for genetic investigation in anaerobes [65]. The main difficulty was to transfer recombinant plasmids into anaerobe recipients. The transformation of *C. acetobutylicum* protoplasts with plasmid DNA was an efficient method, but the regeneration of protoplasts into viable bacteria was problematic. Finally, electroporation was a more suitable method of transformation in anaerobes. An additional insight into antibiotic resistance concerned the 5-nitroimidazole, which is widely used in the treatment of infections caused by anaerobes. However, some *B. fragilis* strains are resistant to this antibiotic. The mechanism of resistance to 5-nitroimidazole has been elucidated, and the genes (*nim*) responsible for the resistance have been characterized [66,67].

M. Sebald contributed actively to the investigation of human botulism in France. In total, 660 cases were confirmed in the period from 1970–1996. In 1974, the Laboratory of Anaerobes was recognized as the National Reference Center (NRC) of Anaerobes including the survey of botulism and was renamed the NRC of Anaerobes and Botulism in 2002. Since 1973, the laboratory’s diagnosis of botulism has been improved by the detection of the toxin in the serum of patients [68]. Until this period, the confirmation of botulism was only based on an investigation of suspected foodstuffs but only when they were available. In the 1990s, the first identifications of botulism outbreaks in waterbirds and farmed birds due to *C. botulinum* type C were performed in France [69]. No incidence of the increasing cases of avian botulism on human health was reported.

In the 1980s, the emergence of *C. difficile* antibiotic-associated diarrheas and pseudomembranous colitis prompted the NRC of Anaerobes to initiate the diagnosis of these infections in France by toxin detection based on an assay for cytotoxicity in patient’s feces and to develop bacteriological investigations of this pathogen. In 2008, a laboratory on *C. difficile* managed by Bruno Dupuy was created and was mainly involved in the regulation of toxinogenesis and the genetic aspects of this pathogen (see the article of B. Dupuy in this issue).

Bernard Bizzini joined the service of Immunochemistry managed by M. Raynaud in the annex of Garches in 1952 and supported his PhD on TeNT in 1970. In collaboration with A. Turpin and M. Raynaud, B. Bizzini developed the production and purification of TeNT [58]. Then, B. Bizzini moved to the Institut Pasteur, Paris, to the department of Protein Chemistry in 1974 and then to the unit of Protein Immunochemistry (1980–1992) where his main activity was in TeNT. B. Bizzini contributed to deciphering the TeNT structure by a biochemical approach based on dissection by proteases and in determining the biological and immunological properties of TeNT fragments [38,70]. Thus, in collaboration with Klaus Stoeckel and Manfred Schwab, he showed that a fragment of the TeNT heavy chain mediates the binding to ganglioside and retrograde axonal transport [71]. Through multiple collaborations, B. Bizzini participated in the elucidation of the TeNT structure function.

Joseph Alouf (1929–2014), was born in Lebanon and has performed his pharmaceutical and scientific studies in Paris. In 1956, J. Alouf joined the service of Bacterial Chemistry at Garches that was managed by M. Raynaud in the Service des Anaérobies of A.-R. Prévot. His interest was in the investigation of hemolysins. He first analyzed the physicochemical and biological properties of streptolysin O from *Streptococcus pyogenes*, and then he investigated related hemolysins such as perfringolysin from *C. perfringens* and listeriolysins from *Listeria* sp. which are now known as the family of cholesterol-dependent cytolysins. In 1972, he moved to the Institut Pasteur Paris as the head of the Laboratory of Bacterial Toxins within the Laboratory of Anaerobes directed by M. Sebald. In 1977, a novel laboratory space was attributed to J. Alouf, and he was promoted to head of the Bacterial Antigens unit, which became the unit of Microbial Toxins in 1992. J. Alouf combined toxinology and immunology, notably regarding bacterial toxins as superantigens. Besides his research activity, he was involved in teaching in particular as the director of the General Immunology course at the Institut Pasteur for 20 years [72]. In addition to his scientific articles, J. Alouf was an editor of several books on bacterial toxins such as the Comprehensive Sourcebook of Bacterial Protein Toxins (four editions between 1991 and 2015) [73].

Numerous scientists joined or visited the Bacterial Antigens/Microbial Toxins unit. This period, from about 1980 to 2000, which witnessed the genetic characterization and discovery of the molecular mode of action of many bacterial toxins was very exciting. Michele Mock and her group investigated the genetics of *Bacillus anthracis* and anthrax toxins, notably the edema toxin that is an adenylate cyclase [74,75] (see the article of Pierre Goossens in this issue). Patrice Boquet performed his postdoctoral fellowship in the laboratory of Alwin Max Pappenheimer, Harvard University, Boston, which played a central contribution in the discovery of the enzymatic activity of the diphtheria toxin ADP-ribosylation of the elongation factor 2 leading to an inhibition of protein synthesis. P. Boquet was interested in the mode of entry of the diphtheria toxin into cells. In 1978, he joined J. Alouf’s group at the Institut Pasteur. In addition to his work on diphtheria toxin entry into cells and the genetic characterization of the diphtheria toxin gene [76,77], he showed that the hydrophobic N-terminal domain of the TeNT heavy chain forms channels into lipid membranes at acidic pH [78,79]. Thus, his research supported the notion that the diphtheria toxin and TeNT use a similar entry pathway into cells based on pore formation through the endosomal membrane at acidic pH. In 1985–1986, Michael Gill a fellow of the Papenheimer’s group who discovered the ADP-ribosylating activity of the cholera toxin [80], came to the Institut Pasteur for a sabbatical year. His project was to analyze the ADP-ribosylating activity of *C. botulinum* C and D. Indeed, Klauss Aktories and colleagues demonstrated that C2 toxin from *C. botulinum* C ADP-ribosylates cellular actin [81]. As the already identified ADP-ribosylating toxins at this time recognized a G-protein (GTP-binding protein) as a substrate, M. Gill suspected that a functional substrate of the C2 toxin was a G-protein, probably of small size, but not actin that is an ATP-binding protein. Experiments performed at the Institut Pasteur showed that supernatants of *C. botulinum* C and D exhibited ADP-ribosylating activity towards a small molecule of about 21 kDa in addition to actin. However, the p21 ADP-ribosylation was not due to the C2 toxin but to a distinct toxin or enzyme called C3. K. Aktories, who was aware of the scientific activity at the Institut Pasteur, requested a *C. botulinum* D strain from M. Sebald and promptly published the ADP-ribosylation of the C3 enzyme [82]. Then, the group of Narumiya identified the substrate of the C3 enzyme as a small G-protein homologous to Ras protein called Rho (Ras-homologous) [83]. Pierre Chardin, P. Boquet, M. Gill, et al. showed that C3 through the ADP-ribosylation of Rho induces a disorganization of the actin cytoskeleton [84]. Later, Alan Hall’s group demonstrated that Rho and related G-proteins (Rac, Cdc42) are key molecules involved in the regulation of actin cytoskeleton structures [85,86]. Thereby, the C3 enzyme, which is specific to the Rho protein, is a powerful tool in cell biology and was intensively used to unravel the regulation of actin cytoskeleton assembly.

The discovery of the C3 enzyme prompted research to identify other possible ADP-ribosylating toxins in clostridia. Thus, many clostridial strains have been tested, and other toxins from the C2 family were identified such as *Clostridium spiroforme* toxin and *C. difficile* transferase (CDT) [87,88]. Interestingly, the *C. difficile* strains which produce CDT in addition to the two large molecular size toxins, toxin A (TcdA) and toxin B (TcdB), and which acquire fluoroquinolone resistance, were recognized as human epidemic strains such as ribotypes 027, 078 [89]. The cloning of the Iota toxin genes from *C. perfringens*, a toxin related to the C2 toxin, revealed a high level of similarity between the binding component of the Iota toxin (Ib) and the protective antigen (PA) of the anthrax toxins [90]. Later, both components were found to share a similar structure and mode of action in pore formation through endosome membranes facilitating the translocation of the enzymatic components into the cytosol [91].

In the late 1980s, the TeNT and BoNT/A genes were cloned and sequenced by the German group of Heiner Niemann [92,93]. In the Bacterial Toxin unit and in collaboration with Niemann’s group, the sequencing of the neurotoxin gene of *C. botulinum* C, D, E, and *C. butyricum* E was been performed (reviewed in [94]). The neurotoxin sequencing revealed that all the TeNT and BoNT types contained a signature of zinc-dependent proteases. The enzymatic activity of clostridial neurotoxins was subsequently characterized by Cesare Montecucco, Giampietro Schiavo, et al. [95]. Then, the first complete botulinum locus including the genes of BoNT and non-toxic proteins which associate with the neurotoxin to form botulinum complexes was characterized in *C. botulinum* C [96]. The role in the neurotoxin synthesis of the regulatory genes (*botR*) from the botulinum loci and *tetR* upstream of the *tent* gene has been investigated. These genes encode for alternative sigma factors that positively control neurotoxin production (review in [94]).

From 1997 to 2019, the Anaerobic Bacteria and Toxins unit at the Institut Pasteur investigated several clostridial toxins (*C. perfringens* epsilon, beta2, delta toxins, *C. sordellii* lethal toxin, *C. difficile* toxins, *C. septicum* alpha toxin) and further characterized clostridial neurotoxins in collaboration, for some aspects, with external scientists such as Klaus Aktories, Holger Barth, Roland Benz, Juan Blasi, Holger Brüggemann, Christian Lévèque, Jordi Molgo, Bernard Poulain, Bradley Stiles, and Richard Titball [97].

In 2019, the unit was renamed Bacterial Toxins and was directed by Emmanuel Lemichez. This unit is dedicated to clostridial neurotoxins and other bacterial toxins such as *Escherichia coli* CNF (see the article of Lemichez on this issue).

## 7. Concluding Remarks

Since the founding of the Institut Pasteur in 1887 for developing a rabies vaccination, microbiology, including research and training, has been a continuous and central purpose of this institute. The microbiology research activity was performed in a common laboratory called the “Microbie technique”. Then, anaerobic bacteriology and toxinology emerged as distinct activities which required specific equipment and were developed in more specialized laboratories. First, taxonomy and the identification of pathogens were the main tasks. Subsequent investigations on the mechanisms of pathogenicity showed that some bacteria secrete soluble toxic factors that are responsible for symptoms and lesions. In-depth analysis of toxins over time according to the development of technology (biochemistry, enzymology, molecular biology, cellular biology, crystallography) brought a better understanding of certain diseases and allowed the development of specific preventions and treatments (vaccines based on anatoxins, serotherapy, toxin inhibitors). Moreover, toxins were found to be efficient therapeutic drugs (for example, therapeutic applications of BoNTs) and also powerful tools in dissecting specific cellular processes. Thus, toxins contribute to the emergence of the concept of cellular microbiology [98]. Throughout its history, the Institut Pasteur has made relevant contributions in anaerobic bacteriology and toxinology.

## Data Availability

Not applicable.
